# Impact of Histological and Molecular Parameters on Prognosis of Oral Squamous Cell Carcinoma: Analysis of 290 Cases

**DOI:** 10.1155/2020/2059240

**Published:** 2020-10-14

**Authors:** B. S. M. S. Siriwardena, H. D. N. U. Karunathilaka, P. V. R. Kumarasiri, W. M. Tilakaratne

**Affiliations:** ^1^Department of Oral Pathology, Faculty of Dental Sciences, University of Peradeniya, Sri Lanka; ^2^Centre for Research in Oral Cancer, University of Peradeniya, Sri Lanka; ^3^Department of Community Medicine, Faculty of Medicine, University of Peradeniya, Sri Lanka; ^4^Department of Oral and Maxillofacial Clinical Sciences, Faculty of Dentistry, University of Malaya, Malaysia

## Abstract

**Background:**

Nodal metastasis is a critical factor in predicting the prognosis of oral squamous cell carcinoma (OSCC). When patients present with a clinically positive neck, the treatment of choice is radical neck dissection. However, management of a clinically negative neck is still a subject of significant controversy.

**Aim:**

This study was carried out in order to propose a model to predict regional lymph node metastasis of OSCC using histological parameters such as tumour stage, tumour size, pattern of invasion (POI), differentiation of tumour, and host immune response, together with the expression levels of six biomarkers (periostin, HIF-1*α*, MMP-9, *β*-catenin, VEGF-C, and EGFR), and, furthermore, to compare the impact of all these parameters on recurrence and 3 yr and 5 yr survival rates. *Materials and Method*. Histological materials collected from the archives were used to evaluate histological parameters and immunohistochemical profiles. Standard methods were used for immunohistochemistry and for evaluation of results. Data related to recurrence and survival (3 and 5 years) was also recorded. Clinical data was collected from patients' records.

**Results:**

Male to female ratio was 3 : 1. The commonest site of OSCC was the buccal mucosa, and majority of them were T3 or T4 tumours presented at stage 4. 62.5% of the tumours were well differentiated. Three-year and 5-year survival rates were significantly associated with lymph node metastasis and recurrence. POI was significantly correlated with tumour size, stage, 3-year survival, EGFR, HIF-1*α*, periostin, and MMP-9 (*p* < 0.05). Expression of EGFR showed a direct association with metastasis (*p* < 0.05).

**Conclusion:**

POI, level of differentiation, and expression of EGFR are independent prognostic markers for lymph node metastasis. Therefore, these parameters may help in treatment planning of a clinically negative neck.

## 1. Introduction

Oral cancer is the commonest cancer in men in Sri Lanka, and 3-4 people die every day due to the disease [1]. Nodal metastasis is a significant prognostic indicator. When patients present with a clinically positive neck, the treatment of choice is excision of the tumour with radical neck dissection (RND), whilst the management of a clinically negative neck is still controversial. Usually, for the latter, either elective neck dissection (END) or “watchful-waiting” policy is practised [2]. Although END could help in eliminating undetectable metastasis, majority of the patients may not need such radical treatment, as it is associated with significant morbidity. In addition, it removes the natural barrier, the lymphatic system [3, 4].

Size and primary site of the tumour, TNM stage, and some histopathological parameters have a significant impact on prognosis. Tumour differentiation, pattern of invasion, tumour thickness, host immune response, and vascular and perineural invasion are some of the proven parameters. We and others have demonstrated with previous studies that the pattern of invasion is a very reliable factor in predicting metastasis [5–7].

Invasive front or the tumour-host interface of the cancer is known to be important in predicting metastasis and prognosis [8]. Many studies have proven that the location of molecular markers at the invasive front is more important than assessment of their expression in the whole tumour [9]. The number of studies assessing the invasive front using molecular markers appeared to be inadequate to draw scientifically valid conclusions to manage patients [3]. Therefore, analysis of the expression levels of various molecules at the invasive front in a larger sample of patients is a necessity to identify the markers that predict metastasis and prognosis.

In our previous studies, we have shown that there is a significant expression of molecular markers such as hypoxia-inducible factor-1*α* (HIF-1*α*) [10, 11], periostin [12], matrix metalloproteinase-9 (MMP-9) [13], vascular endothelial growth factor-C (VEGF-C) [14, 15], epidermal growth factor receptor (EGFR) [16], and *β*-catenin [16] in OSCC and their ability to serve as potential molecules for predicting metastasis. Further, it has been stated that the use of molecular markers, together with traditional histological parameters, improves comprehensive management of patients with OSCC [17]. This may provide a better prediction of prognosis and proper guidance for management of neck nodes. This will invariably reduce the morbidity and cost of advanced treatment procedures.

Although the significance of many of the biomarkers in OSCC has been reported, large prospective studies integrating the most reliable predictive biomarkers are crucial to control the variability and for definite clarification of the prognostic significance of some of these molecular markers [17]. Therefore, the present study is aimed at investigating the expression of the abovementioned molecular markers at the invasive front and at comparing their relationship with histological parameters, metastasis, and survival. This has not been widely studied using a large number of patients. We hypothesize that the above markers in different combinations and selected histological parameters may have a role in predicting metastasis, hence the survival.

Therefore, our aim was to establish a prediction model combining histopathological parameters with a few selected molecular markers.

## 2. Materials and Methods

### 2.1. Case Selection

Histologically confirmed OSCCs treated with excision of the lesion and neck dissection at different oral and maxillofacial units of Sri Lanka from January 1999 to December 2013 were selected for the study. Demographic and clinical data were gathered from clinical records of patients, and histological information such as tumour differentiation and nodal status were obtained from the oral pathology database. Survival and recurrence data were also collected.

### 2.2. Histopathological Assessment

Paraffin-embedded tissue blocks and slides of the primary tumours and the metastatic nodes were retrieved. The best representative section from the main tumour was selected to assess the pattern of invasion, and the same paraffin block from all cases was used to take sections for immunohistochemical staining. First and last slides were stained with haematoxylin and eosin to confirm the presence of tumour. Data pertaining to nodal status was recorded for each level. The cases with inadequate data were excluded from the study.

### 2.3. Immunohistochemistry (IHC)

The sections were placed on APS-coated slides. The tissue sections were deparaffinized and rehydrated in a graded series of alcohols. Endogenous peroxidase activity was blocked with 0.3% H_2_O_2_ in methanol for 30 minutes. All selected cases were stained by the EnVision+ system using primary antibodies for EGFR [18], *β*-catenin [19], VEGF-C [14], MMP-9 [20], periostin [12], and HIF-1*α* [11]. Incubation time for each antibody is stated in Table 1.

The immunohistochemical staining was evaluated with a grading system according to the intensity of staining. Staining was recorded for each antibody on each case independently, especially at the advancing front (Table 2).

### 2.4. Statistical Analysis

Statistical analysis was performed using SPSS for Windows version 16. Data was analysed using descriptive statistics. Pearson's chi-square test was used to compare proportions. The statistical significance was accepted at *p* < 0.05. Logistic regression was used to determine a model for metastasis of OSCC with the histological parameters and molecular biomarkers.

## 3. Results

### 3.1. Demographic Data

#### 3.1.1. Sex

Total of 290 cases were selected for the study. Of them, 218 were males and 72 were females (male : female, 3 : 1) and age ranges from 31 to 85 years. Majority of the patients were in the 51-60-year age group (34.8%). The commonest site was the buccal mucosa for both males and females (45% and 44.4%, respectively). However, majority of tongue cancers were in the 31-40-year age group (54%). Most tumours were at T3 (48.6%) and stage 4 (57.1%) irrespective of the gender.

Both males (49.5%) and females (52.8%) had mainly well-differentiated SCC. Moderate host immune response was commonly seen in both genders (47.7% in males and 48.6% in females). However, most OSCCs with dense host immune response of 29.2% were recorded among females. Pattern of invasion type IV was found to be the commonest among males with 40.8%, and it was 37.5% in females.

The overall 3-year and 5-year survival rates were 55.1% and 37.3%, respectively. Three-year survival rate for males was 52.8% whilst it was 62.8% for females. Five-year survival was 38.6% and 31.8% in males and females, respectively. 59% of the males and 60.9% of the females had recurrences. Out of the total sample, 36.5% had nodal metastasis although there was a slight preponderance towards females (38.9%).

#### 3.1.2. Age

There was no significant gender difference or primary site of occurrence for different age groups, except that SCC in the tongue was common in younger age groups (31-40 years) accounting for 54% of the total sample. T1 and T2 tumours were mainly seen in the younger group of patients. Among all age groups, majority of the tumours were T3 and stage 4 except in the 31-40-year age group where stage 3 (74.5%) was the commonest. Predominant POI was type IV and the 31-40-year category had the highest percentage of POI type IV (45.5%). Highest 3-year survival rate of 56.8% was found in the 31-40-year age group indicating younger patients had a better 3-year survival. In contrast, 5-year survival was better in the 51-60-year group (51.4%), and all other groups were below 50%. More recurrences were recorded in the 31-40-year group (75.0%), and the same group had higher metastasis (45.5%). Therefore, it was obvious that lymph node metastasis was higher in the younger (<40 years) age group.

#### 3.1.3. Primary Site of OSCC

Buccal mucosa (BM) was the most common primary site (44.8%); hence, most T4 tumours were from the BM. However, majority of the stage 4 tumours were in the lower alveolar ridge (72.2%). Most well-differentiated tumours and tumours with dense immune response were from buccal mucosa while the highest percentage of poorly differentiated tumours (10.9%) was recorded from the tongue.

Majority of lower alveolar ridge SCC had the highest percentage of lymph node metastasis (46.2%) followed by the tongue (41.1%) while buccal mucosa had the highest number (90, 69.2%) of metastasis-negative cases. POI type IV was mainly identified in tongue cancers (65.8%) and least in BM cancers with low metastatic potential. A statistically significant association was identified between the primary site and POI (chi-square value 36.9, *p* < 0.05). 58.8% of the tongue cancer patients survived less than 3 years. The highest percentage of patients who survived >3 years was when the tumour was present on the buccal mucosa and lower alveolar ridge (61.5%). However, the results were not statistically significant. Similarly, the poorest 5-year survival rate was observed from tongue SCC (21.4%). Tumours in the buccal mucosa recorded the highest 5-year survival out of all primary sites (42.9%).

#### 3.1.4. Size and Stage of Tumour

Out of the total sample, T3 tumours were 43.8% and T1 tumours were only 2.3%. Size of the tumour was not available in 160 cases. Although T1 and T2 tumours were mainly seen in the younger group, the results were statistically not significant. Majority of stage 4 tumours (61.1%) were T4. A statistically significant association was observed between these 2 variables (*p* < 0.05). The relationship between tumour size with POI (*p* < 0.05) and nodal metastasis (*p* < 0.05) was also found to be significant statistically.

57.7% of the cases were in stage 4 and 5.2% in stage 1 (data was available in 194 cases). Combination of stages 3 and 4 was almost 80% as most patients seek their treatment at the late stage leading to difficult management and poor prognosis. A statistically significant association was revealed between tumour stage and host immune response (chi-square value 13, *p* < 0.05). Stage of the tumour and POI also showed a statistically significant association (chi-square value 18.9, *p* < 0.05). When metastasis was compared with tumour stage, most of the nodal-positive cases (72.7%) were from stage 4 (chi-square value 10.0, *p* < 0.05).

### 3.2. Histopathological Parameters

#### 3.2.1. Host Immune Response

Host response [5, 8] was moderate in half of the cases (47.9%), and only 7.9% of the cases had no host response. Significant number of lower alveolar ridge tumours (19.2%) showed no host response at all. Least number of lymph node metastasis was identified in the dense response group (chi-square value 8.8, *p* < 0.05). Patients' with dense host response significantly survived for 3 years (72.7%) (chi-square value is 9.7, *p* < 0.05). Survival data was available in 15 cases in the minimal host response category, and survival rate was 26.7%. No significant relationship was observed between 5-year survival rate and host response. Host immune response is an important variable in prognosis of OSCC and included in the logistic regression model to identify which variable affects metastasis of OSCC.

#### 3.2.2. Differentiation of the Tumour

Well and moderately differentiated tumours were the majority (91%) whilst poorly differentiated tumours were the least (5.5%). Out of the total sample, 3.4% of the cases were early invasive squamous cell carcinomas. 62.5% of the nodal-negative cases were well-differentiated tumours whilst 68.8% of the poorly differentiated OSCCs revealed nodal metastasis (chi-square value 32.4, *p* < 0.05). Differentiation of the tumour is an important variable in predicting prognosis of OSCC, and it was included in the logistic regression model.

#### 3.2.3. Nodal Involvement

Lymph node metastasis is one of the major prognostic indicators in OSCC. In the present sample, majority of the cases presented without metastasis (63.4%). Out of the nodal-positive cases, 52.8% showed extracapsular invasion.

Nodal involvement was categorized as negative, positive without extra capsular invasion, and positive with extracapsular invasion. Most cases with extracapsular invasion showed POI type IV (58.0%). Similarly, 50% of capsular invasion cases also had POI type IV. OSCCs that showed POI type II had very low metastatic rate (15.4%) and were significant (chi-square value 24.3, *p* < 0.05). Nodal metastasis and POI also showed a significant relationship (chi-square value 23.2, *p* < 0.05). Further, a statistically significant association was observed between tumour size, stage, host response, tumour differentiation, and EGFR expression with lymph node metastasis (*p* < 0.05) (Table 3).

A statistically significant relationship was found between metastasis and 3-year survival. 66.7% of nodal-negative cases survived for 3 years whilst 33.3% survived for 3 years when lymph nodes were positive (*p* < 0.001). Similarly, of the patients with positive nodes, 15.4% survived for 5 years, and in the negative node group, 50% survived for 5 years (*p* < 0.001). Patients who did not have metastasis had higher survival, and it appears that survival depends on regional lymph node metastasis.

#### 3.2.4. Pattern of Invasion

Pattern of invasion (POI) is a significant predictor of lymph node metastasis. There are 4 grades depending on arrangement of tumour cells at the advancing front ranging from I to IV [5]. Patterns IV (40%) and III (33.1%) were more common than pattern II. There were no cases of pattern I in the sample.

A statistically significant relationship between 3-year survival and POI was observed (chi-square value 17.7, *p* < 0.05). 66.1% of the sample without 3-year survival had POI type IV. Similarly, 68.1% of the patients with POI type IV have not survived for 5 years, and the results were significant statistically (chi-square value 25.3, *p* < 0.05). Majority of the recurred tumours had POI type IV (54.8%) (chi-square value is 7.7, *p* < 0.05). Furthermore, EGFR, MMP-9, HIF-1*α*, and periostin showed a positive correlation with POI. It is evident that POI is extremely important in prognosis of OSCC; hence, it was included in the logistic regression model as a variable affecting metastasis.

### 3.3. Tumour Recurrence

Data related to recurrence were available in 123 cases, and 59.3% had local recurrences. Between 5-year survival and recurrence, a statistically significant association was revealed. 85.4% of the cases with 5-year survival had no recurrence, and 77.9% of the patients without 5-year survival were presented with recurrence of tumour (*p* < 0.05).

### 3.4. Immunohistochemical Markers

#### 3.4.1. EGFR

**Figure 1:**
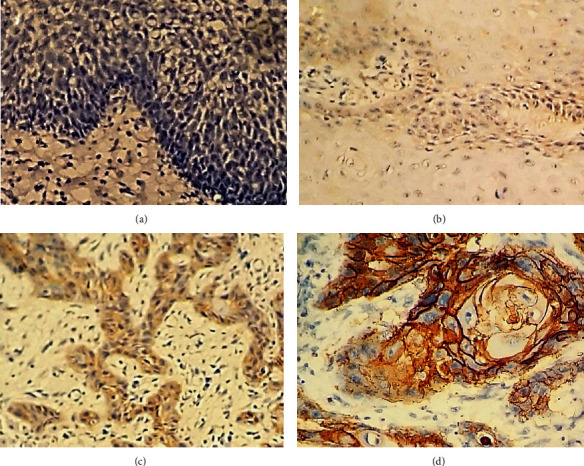
Expression of EGFR in the present study sample. (a) Negative staining of EGFR in normal epithelium. (b) Weak staining of EGFR in OSCC. (c) Moderate staining of EGFR in OSCC. (d) Strong staining of EGFR in OSCC.

EGFR expression can be detected in the cell membrane and cytoplasm of tumour cells (Figure 1). Immunohistochemically, intense expression of EGFR was observed in 78.3% of the cases, and majority of them had POI type IV while majority of weak expression cases showed POI type II (chi-square value 36.8, *p* < 0.05). Furthermore, a significant relationship with metastasis was also observed (*p* < 0.003).

#### 3.4.2. *β*-Catenin

*β*-Catenin expression was detected in the cytoplasm of normal cells and not in cancer cells and was not expressed in majority of the cases (206 out of 290). Only 29.0% of the cases had a positive expression. Majority of POI type IV showed negative *β*-catenin expression. Although the results are not significant, more metastasis was seen in the negative *β*-catenin expression group.

#### 3.4.3. HIF-1*α*

Nuclear and cytoplasmic expression of HIF-1*α* was detected in tumour cells. 75.2% of cases showed strong expression. A significant correlation was found between HIF-1*α* and POI type IV suggesting a relationship with poor prognosis (chi-square value 7.8, *p* < 0.05).

#### 3.4.4. Periostin

Periostin expression was detected in the cytoplasm of cancer cells in all cases. Intense staining was observed in 64.5%. Out of highly expressed tumours, 43.9% had POI type IV and was statistically significant (chi-square value 7.0, *p* < 0.05).

#### 3.4.5. VEGF-C

36.9% of cases showed intense membranous and cytoplasmic positivity for VEGF-C. Majority (45.8%) of the intense VEGF-C expression cases had POI type IV. Most of the metastasis-negative cases (61.9%) had a low expression of VEGF-C.

#### 3.4.6. MMP-9

Strong expression of MMP-9 was detected in 40.2%, and the rest had low expression. A statistically significant association was found between MMP-9 and POI (chi-square value 12.8, *p* < 0.05).

### 3.5. Logistic Regression

A logistic regression statistical model was used to identify the risk factors which are independently responsible for the occurrence of metastasis among the patients. Based on the results of the univariate analysis tumour stage, tumour size, host response, differentiation, POI, and EGFR expression were fitted to the statistical model.

The final regression model identified host immune response, tumour differentiation, and POI as the risk variables which are independently responsible for the occurrence of metastasis. Within the POI, pattern with individual tumour cells (IV) is most significantly associated with the occurrence of metastasis compared to the POI type II (Table 4).

## 4. Discussion

According to the WHO, there is an estimated 657,000 new cases of cancers of the oral cavity and pharynx each year, and more than 330,000 deaths are reported worldwide. The burden is particularly higher in South Asia due to frequent exposure to risk factors such as betel quid chewing, smoking, and alcohol consumption [21, 22]. Sri Lanka has the eighth highest oral cancer mortality rate in the world. Lip and oral cavity cancer is the number one ranked malignancy among men and 5^th^ in women in Sri Lanka [1, 23].

More than 90 out of 100 (90%) mouth and oropharyngeal cancers are squamous cell carcinomas [24]. Even though the disease burden is as per the situation described earlier, a very minimal number of studies have been carried out in Sri Lanka to identify the connection between histopathological and molecular parameter expressions with prognosis of OSCC. Three main categories such as patient, tumour, and treatment-related factors are important in predicting the prognosis of OSCC [3]. Sex and age, tobacco and alcohol, socioeconomic conditions, and diagnostic delays were identified as patient-related factors while anatomic site, disease staging, cervical node metastases, depth of invasion, extracapsular spread, histological differentiation, and molecular markers were described as tumour-related factors.

Therefore, we focused on some of the patient- and tumour-related factors, such as demographic data, anatomic site, tumour stage, lymph node metastasis, POI, histological grading, and expression of selected molecular markers. Although many studies have attempted to analyse various combinations of these factors, a model with reliable practical implications for the management of OSCC is yet to be developed [25–27].

### 4.1. Distribution of Demographic Data

According to national cancer incidence data 2014, the highest overall cancer incidence was recorded from the age category 70–74 years with 571.7 per 100,000 in Sri Lanka [1]. In a study on independent prognostic factors of 861 cases of OSCC in Korean adults, a multivariate Cox regression analysis has shown that age, gender, composite stage, and treatment method were significant independent prognostic factors [28]. Oral cancer is usually a disease that occurs in males after the 5th decade of life [29]. Coinciding with this observation, in the current study, out of a sample of 290, the highest number of patients was from the 51–60-year category. But age was not identified as an independent prognostic factor. However, a retrospective study in Karachi, Pakistan, had 77.9% (out of 80 OSCCs) within the 31-60-year age group [30].

The lip, oral cavity, and pharynx are the leading cancer sites for Sri Lankan males (24% of all cancer sites). According to national cancer statistics, 1888 cases were reported in males, while 534 cases among the females [1]. Therefore, the average male to female ratio of oral cancer is 3.5 : 1. According to a study on epidemiology and aetiology of OSCC worldwide, females are less commonly affected by oral cancer, largely reflecting greater use of relevant habits by men [29, 31]. Our results were similar to these findings with 75.2% cases in males (male : female = 3 : 1), confirming the predominance of OSCC in males in Sri Lanka. The prevalence of OSCC due to HPV infection has caused a change in gender distribution [32].

### 4.2. Clinical and Histopathological Parameters

According to the studies by Mirza D et al. [30] and Mirza S et al. [33], buccal mucosa was encountered with the highest percentage of OSCC (50% and 46.8%, respectively). Another study also showed that most tumours were seen in the buccal cavity (54%) followed by the tongue (24%) [34]. In contrast, a Brazilian study showed that 37% of cases out of 346 were from the lateral border of the tongue [35]. In an Indian study with 295 OSCC cases, the most common site was the mandibular alveolus region [36]. In the current study, the buccal mucosa was the most common primary site in both genders. However Sahaf et al. reported that majority of females (41.7%) in their study had SCC in the tongue, while majority of males (33.3%) had SCC in the buccal mucosa [31]. Hence, it is evident that the most common site of OSCC changes from population to population. The main reason for this site predilection in different regions is due to risk habits. In countries in South Asia where betel quid chewing is common, the frequent site of occurrence is the buccal mucosa as they keep the quid in the buccal pouch. In Western countries where alcohol and smoking are common practices, the frequent sites of involvement are the tongue and floor of the mouth. Apart from these main habits, factors such as genetic predisposition, viruses, and radiation can be considered as other reasons for this difference.

Early detection of cancer is very vital in the prognosis of patients identified with OSCC. The survival rates highly rely on the stage of the tumour at diagnosis. 57.7% of the cases in this study presented to clinicians at stage 4 and only 5.2% at stage 1. Mirza et al. also indicated that their stage 4 and stage 1 presentations were 50.7% and 14.7%, respectively, and they found a significant association with stage and metastasis [33]. In an Indian study, majority of the patients (243, 82.37%) were presented at stage 3 and none at stage 1 [36]. Another study in Sri Lanka, where 193 previously diagnosed OSCC patients were followed up for 5 years, found that the tumour stage has a significant association with the 5-year survival (*p* < 0.05) [6]. The current study found a statistically significant association between stage and metastasis (*p* < 0.05).

A positive significant correlation between the host response and metastasis of OSCC was found by logistic regression analysis. Our above results are in agreement with the findings by de Matos et al. [37]. In a study by Chatzistamou et al. [38] on oral tongue SCC, high-density inflammatory host response was correlated with a favourable survival. Lundqvist et al. [39] found a correlation between density of host response and favourable response to radiotherapy in tongue SCC. These results were in line with our study, where a statistically significant association between the 3-year survival rate and host response was revealed (*p* < 0.05). Therefore, it can be assumed that the antitumour characteristics of inflammatory cells might be a reason for better prognosis of patients.

In this study, most of the tumours were well differentiated (50.3%) similar to the findings by Sahaf et al. [31] where 68.4% of tumours were well differentiated. However, Lundqvist et al. [39] and Mirza et al. [33] showed in their studies that the majority were moderately differentiated tumours (40% and 62.1%, respectively). Logistic regression analysis showed a positive relationship between metastasis and tumour differentiation where metastatic rate was increased when tumour differentiation varied from well to poor. In a study done by Dissanayaka et al. [6], degree of differentiation was well correlated with the five-year survival rate. Further, it is advisable to include level of differentiation to multivariable models of survival as a covariate to improve prognostic accuracy [40].

The cervical lymph node status is considered the most important prognostic factor in head and neck cancer. According to a study by Woolgar et al. [41], microscopic extracapsular spread is of critical importance and it should be incorporated into pathological staging systems. In the current study, a statistically significant relationship was found between metastasis and 5-year and 3-year survival rates (*p* < 0.05). Some studies showed a significant association between POI and metastasis [42, 43], and this study also revealed the same.

POI has been identified as one of the most reliable predictive factors of OSCC. POI type IV where there is an individual tumour cell invading the host tissues at the tumour front suggests poor prognosis [6]. Majority of the patients who did not survive for 3 years and 5 years had POI type IV (*p* < 0.05), and many other studies supported this finding [44–46]. Furthermore, our study showed that most (70.5%) of the POI type IV was from stage 4 cases and may be served as a contributory factor for poor prognosis. However, the available literature has not analysed the above relationships. Extracapsular spread and POI showed a positive correlation. Majority of the younger patients in the present study also had POI type IV thus leading to poor prognosis.

The time span to distinguish the lesion as a recurrence (local recurrence/nodal recurrence) has not been well-described. Camisasca et al. [47] have demonstrated that poorly differentiated tumours located in the tongue and showing POI type III or IV are associated with higher recurrence. Majority of the cases that presented with recurrence in our study were from POI category IV (*p* < 0.05). According to the study carried out by Brandwein-Gensler et al. [48], the traditional POI in the Bryne et al. [49] multifactorial grading system at the tumour front has been modified by adding a pattern V which is defined as tumour satellites, and this parameter is identified as worst POI (WPOI) at the tumour/host interface. This WPOI has been identified as an independent predictor of local recurrence and overall survival. Recurrence is considered the most common cause of treatment failure and is usually attributed to incomplete surgical resection of the tumour [50]. The highest recurrence percentage of 75.0% was reported from the age 31–40-year category in the current study. This confirms that the presence of type IV POI in the younger group leads to high recurrence, hence poor prognosis with reduced 5-year survival rate.

Survival of the cancer patient is the main prognostic indicator of OSCC. Despite recent advances in treatment, the 5-year survival rate of OSCC still remains at 50% [51]. Locoregional recurrence has been identified as an important factor that affects survival [48]. Based on the Kaplan-Meier survival analysis, Yanamoto et al. [46] revealed similar results to current findings. In their study, 5-year cancer-specific survival rate was 92% in the nonrecurrence group and 30% in the recurrence group (*p* < 0.0001, log-rank test). Comparatively, Wang et al. [52] using the Kaplan-Meier method and log-rank test showed that 2-year and 5-year survival rates were lower in patients with recurrence than in those without recurrence (67.6% vs. 88.0%, 31.8% vs. 79.9%, *p* < 0.001). Camisasca et al. [47] also has shown that local recurrence is a significant prognostic factor for 5-year disease-specific survival similar to our study.

### 4.3. Expression of Molecular Markers

The effects of activation of EGFR in keratinocytes include induction of cellular proliferation, reduction in differentiation, induction of migration of normal keratinocytes, and inhibition of apoptosis while increasing survival of keratinocytes. Hiraishi et al. [53] have shown that out of 52 patients with OSCC, 33 had high EGFR expression immunohistochemically (63.4%), and a study on tissue microarray-based IHC by Laimer et al. [54] showed similar results with high EGFR expression of 73.42% of the tumour sample. Statistically significant relationship between lymph node metastasis and survival rates with higher expression of EGFR was identified in the present study (*p* < 0.05). A multivariate analysis by Laimer et al. identified EGFR overexpression as an independent prognostic marker (*p* = 0.02) [54] similar to our study. However, the study by Kudo et al. [55] did not find a positive correlation by Fisher's exact test. This is the first study comparing POI and EGFR expression, and we found a significant association (*p* < 0.05). However, Ang et al. [56] has revealed that immunohistochemical EGFR expression was a strong independent prognostic indicator for overall survival and disease-free survival through a multivariate analysis performed using Cox proportional hazard model. Based on these results, they have suggested that the patients who have EGFR-positive immunoexpression in their OSCCs should be considered for more aggressive combined therapies or enrolment into trials targeting EGFR signalling pathways.

In the present study, EGFR and MMP-9 were found as independent predictive variables in the logistic regression analysis.

Many cancers have been related with the overexpression and mutations of *β*-catenin. Abnormalities of cell adhesion molecules are known to play an important role in invasion and metastasis of cancer cells through loss of cell-to-cell adhesion [55]. A study done by Kudo et al. [55] suggests that the invasion and metastasis of OSCC cells require methylation of E-cadherin and/or degradation of membranous *β*-catenin. In a study with 30 OSCC samples, [57] suggested that the use of loss of expression of *β*-catenin as a marker (by IHC) of nodal metastasis in OSCC is unreliable (Fisher's exact test). However, they have found that loss of expression of *β*-catenin has an association with lower degree of differentiation. Another study using 24 OSCCs showed that there is no difference between the expression of *β*-catenin in metastatic and nonmetastatic groups through nonparametric Mann-Whitney *U* test for exploratory descriptive analysis [58]. Contrasting to these outcomes, Tanaka et al. [16] have found significantly greater reduction in expression level of *β*-catenin in the lymph node metastatic group (*n* = 64) compared to the nonmetastatic group (*n* = 95) (*p* = 0.001) in a study on 159 OSCC samples, using Kaplan-Meier method log-rank test for statistical analysis. Although the results are not significant, more metastasis was seen in the negative *β*-catenin expression group.

HIF-1*α* is a molecule that is mainly activated under hypoxic conditions and has been proposed to play a role in malignant transformation of OSF and may be possible to use as a marker [10, 11]. Santos et al. [59] have demonstrated by IHC on 66 tissue microarrays that high HIF-1*α* expression was associated with local disease-free survival (*p* = 0.013) through chi-square and Fisher exact test and multivariate logistic regression. And also, a multivariate Cox regression analysis suggested that HIF-1*α* is an independent prognostic marker correlating with a disease-specific survival (*p* = 0.001) [60] by IHC on 82 OSCC cases. In the current study, no statistically significant relationship was found between HIF-1*α* and metastasis. However, highly expressed HIF-1*α* cases were from the POI type IV revealing a statistically significant relationship (*p* < 0.05). Although we were unable to find studies connecting HIF-1*α* expression with POI, the relationship we revealed may show an indirect association between HIF-1*α* expression and lymph node metastasis as POI type IV is significantly associated with lymph node metastasis. Therefore, this molecule may have a role as a prognostic marker in OSCC.

Periostin is a secreted protein that stimulates metastatic growth by promoting cancer cell survival, invasion, and angiogenesis; thus, it can be a useful marker to predict the behaviour of cancer (Kudo et al., 2007). In the current study, periostin was strongly expressed in 64.5% of the cases, and yet, we could not demonstrate a statistically significant relationship between periostin expression and metastasis.

Lymphangiogenesis is reported to be induced by vascular endothelial growth factor-C (VEGF-C). This molecule promotes tumour invasion and metastasis. The density of microvessels is increased by VEGF-C influencing the metastatic behaviour of the tumour. In an IHC study, Sedivy et al. [15] concluded that VEGF-C expression in OSCC triggers lymphatic angiogenesis, which may result in a higher risk for cervical lymph node metastasis. Kudo et al. [61] also have identified that periostin expression would induce upregulation of VEGF-C, which may in turn promote lymphangiogenesis. Although majority of high VEGF-C expression cases were from the POI type IV, a statistically significant association was not identified between these two parameters as in the study done by Siriwardena et al. [14]. The current study also did not identify a statistically significant relationship. In contrast to our study, Kishimoto et al. revealed a significant relationship between VEGF-C expression and lymph node metastasis (*p* < 0.001) with Fisher's exact test [62].

Matrix metalloproteinase-9 (MMP-9) is a protein from the matrix metalloproteinase family which plays many roles in the normal physiological processes. Although its role is not clear in cancer, many studies have reported the importance of MMP-9 expression in OSCC [20, 63, 64]. Zhou et al. [64] found a significant difference between MMP-9 overexpression and nodal metastasis (*p* < 0.01). A study done by Ruokolainen et al. [20] showed that MMP-9 expression (IHC) was statistically correlated with cause-specific survival by Kaplan-Meier analysis (*p* = 0.013). Hong et al. also supported the same [63]. However, a statistically significant association was shown between MMP-9 expression and POI in the current study, hence metastasis.

### 4.4. Logistic Regression Model in Multivariate Analysis

When considering the factors that influence metastasis in the current study, logistic regression analysis has been used to specify positive and negative parameters. According to the results, POI and differentiation of the tumour are independent prognostic markers for lymph node metastasis. Although some of the previous studies have discovered a correlation between metastasis of OSCC and some molecular markers [5, 12, 16, 53, 63, 65], we found that EGFR shows a positive correlation. Moreover, none of these studies include a sample size as large as the current study.

High expression of EGFR, HIF-1*α*, periostin, and MMP-9 and loss of expression in *β*-catenin showed a direct relationship with pattern of invasion. As there is a significant relationship between POI with lymph node metastasis and survival, it may indirectly relate the above molecules with lymph node metastasis and survival.

A study with a cohort of 193 patients has revealed tumour stage, POI, and excision margins to be the most predictive factors of survival in OSCC [6]. Pattern of invasion was identified as the best prognosticator in the stepwise Cox regression model [65]. Yuen et al. indicated that tumour thickness was the only significant prognostic factor for subclinical nodal metastasis in a multivariate logistic regression analysis on stage I and II oral tongue carcinomas [66]. They also have shown that both tumour thickness and perineural infiltration were significant independent prognostic factors of local recurrence.

## 5. Conclusions

Three-year and 5-year survival rates were significantly associated with lymph node metastasis and recurrence. POI was significantly correlated with tumour size, stage, 3-year survival, and all the tested molecules. POI, level of differentiation, and expression of EGFR are independent prognostic markers for lymph node metastasis. Therefore, these parameters may help in treatment planning of the clinically negative neck.

## Figures and Tables

**Table 1:** List of antibodies used.

Antibody	Species/clonality	Dilution	Incubation time	Source
Anti-EGFR antibody clone DAK-H1-WT code M7298	Monoclonal mouse antihuman wild-type EGFR	1 : 200	1 hr at R.T	Dako
Anti-*β*-catenin antibody clone *β*-catenin-1 code M3539	Monoclonal mouse antihuman *β*-catenin	1 : 50	1 hr at R.T	Dako
Antiperiostin ab14041	Rabbit polyclonal to periostin	1 : 450	Overnight at 4°C	Abcam
Anti-HIF-1*α* [1A3] ab113642	Mouse monoclonal [1A3] to HIF-1*α*	1 : 400	1 hr at R.T	Abcam
Anti-VEGFC ab135506	Rabbit polyclonal to VEGFC	1 : 100	1 hr at R.T	Abcam
Anti-MMP-9 [EP1255Y]	Rabbit monoclonal [EP1255Y] to MMP-9	1 : 300	2 hrs at R.T	Abcam

**Table 2:** Evaluation of immunohistochemistry.

Molecular marker		Referenced evaluation	Description	
1	EGFR	Hiraishi et al. [53] method Internal (+) control—a known case of OSCC	Extent score (ES)	Intensity score(IS)
			0: negative staining	1: weak staining
			1: <10%	2: moderate
			2: 10%-30%	3: strong
			3: 30%-50%	TES+IS = total expression score (TES)
			4: 50%-80%	
			5: >80%	
			TES: 1-4 > positive expression, 5-7 > high expression	
2	*β*-Catenin	Modified Balasundaram et al. [67] method Internal (+) control—normal oral tissue	Proportion score (PS)	Intensity score (IS)
			0: negative staining	1: weak staining
			1: <10%	2: moderate
			2: 10%-50%	3: strong
			3: 50%-80%	PS × IS = TES
			4: >80%	
			TES: 0 > no expression, 1-12 > positive expression	
3	HIF-1*α*	Modified Santos et al. [59] method Internal (+) control—a breast cancer case	Proportion score (PS)	Intensity score (IS)
			0: negative staining	1: weak staining
			1: <10%	2: moderate
			2: 10%-50%	3: strong
			3: 50%-80% 4: >80%	PS × IS = TES
			TES: 1-7 > positive expression, 8-12 > high expression	
4	Periostin	Modified Kudo et al. [61] method Internal (+) control—gingival cancer cases with periodontal tissue	Proportion score (PS)	Intensity score (IS)
			0: negative staining	1: weak staining
			1: <10%	2: moderate
			2: 10%-50%	3: strong
			3: 50%-80%	PS × IS = TES
			4: >80%	
			TES: 0-4 > low expression, 5-12 > high expression	
5	VEGF-C	Naruse et al. [68] method Internal (+) control—a skeletal muscle section	Proportion score (PS)	Intensity score (IS)
			0: negative staining	1: weak staining
			1: <10%	2: moderate
			2: 10%-50%	3: strong
			3: 50%-80%	PS × IS = TES
			4: >80%	
			TES: 0-4 > low expression, 5-12 > high expression	
6	MMP-9	Modified Sauter et al. [69] Internal (+) control—method	Proportion score (PS)	Intensity score (IS)
			0: negative staining	1: weak staining
			1: <10%	2: moderate
			2: 10%-50%	3: strong
			3: 50%-80%	PS × IS = TES
			4: >80%	
			TES: 0-4 > low expression, 5-7 > moderate expression, and 8-12 > high expression	

**Table 3:** Summary data of the univariate analysis with regard to metastasis.

Variable		Metastasis		Total (%)	*p* ^∗^ value
		Positive (%)	Negative (%)		
Age	31–39 yrs	5 (4.7)	6 (3.3)	11 (3.8)	0.73
	40–49 yrs	17 (16.0)	30 (16.3)	47 (16.2)	
	50–59 yrs	40 (37.7)	61(33.2)	101 (34.8)	
	60–69 yrs	34 (32.1)	58 (31.5)	92 (31.7)	
	≥70 yrs	10 (9.4)	29 (15.7)	39 (13.4)	
	Total	106 (100.0)	184 (100.0)	290 (100.0)	
Gender	Female	28(26.4)	44 (23.9)	72 (24.8)	0.635
	Male	78 (73.6)	140 (76.1)	218 (75.2)	
	Total	106 (100.0)	184 (100.0)	290 (100.0)	
Primary site	Buccal mucosa	40 (37.7)	90 (48.9)	130 (44.8)	0.285
	Tongue	30 (28.3)	43 (23.4)	73 (25.2)	
	lower alveolar ridge	12 (11.3)	14 (7.6)	26 (9.0)	
	Floor of mouth	7 (6.6)	16 (8.7)	23 (7.9)	
	Other	17 (16.0)	21 (11.4)	38 (13.1)	
	Total	106 (100.0)	184 (100.0)	290 (100.0)	
Tumour stage	1 and 2	18 (27.3)	64 (50.0)	82 (42.3)	0.002^∗^
	3 and 4	48 (72.7)	64 (50.0)	112 (57.7)	
	Total	66 (100.0)	128 (100.0)	194 (100.0)	
Tumour size	T1, T2	27 (48.2)	56 (75.7)	83 (63.8)	0.001^∗^
	T3, T4	29 (51.8)	18 (24.3)	47 (36.2)	
	Total	56 (100.0)	74 (100.0)	130 (100.0)	
Host response	Dense	15 (14.2)	50 (27.2)	65 (22.4)	0.01^∗^
	Light	91 (85.8)	134 (72.8)	225 (77.6)	
	Total	106 (100.0)	184 (100.0)	290 (100.0)	
Differentiation	Well	31 (29.2)	115 (62.5)	146 (50.3)	<0.001^∗^
	Poor	75 (70.8)	69 (37.5)	144 (49.7)	
	Total	106 (100.0)	184 (100.0)	290 (100.0)	
Pattern of invasion	POI II	12 (11.3)	66 (35.9)	78 (26.9)	<0.001^∗^
	POI III	37 (34.9)	59 (32.1)	96 (33.1)	
	POI IV	57 (53.8)	59 (32.1)	116 (40.0)	
	Total	106 (100.0)	184 (100.0)	290 (100.0)	
EGFR expression	Strongly positive	93 (87.7)	134 (72.8)	227 (78.3)	0.003^∗^
	Weakly positive	13(12.3)	50 (27.2)	63 (21.7)	
	Total	106 (100.0)	184 (100.0)	290 (100.0)	
*β*-Catenin	No	77 (72.6)	129 (70.1)	206 (71.0)	0.647
	Yes	29 (27.4)	55 (29.9)	84 (29.0)	
	Total	106 (100.0)	184 (100.0)	290 (100.0)	
HIF-1*α*	Positive	24 (22.6)	48 (26.1)	72 (24.8)	0.513
	High	82 (77.4)	136 (73.9)	218 (75.2)	
	Total	106 (100.0)	184 (100.0)	290 (100.0)	
Periostin	Low	38 (35.8)	65 (35.3)	103 (35.5)	0.929
	High	68 (64.2)	119 (64.7)	187 (64.5)	
	Total	106 (100.0)	184 (100.0)	290 (100.0)	
VEGF-C	Low	69 (65.1)	114 (62.0)	183 (63.1)	0.594
	High	37 (34.9)	70 (38.0)	107 (36.9)	
	Total	106 (100.0)	184 (100.0)	290 (100.0)	
MMP-9	Low	61 (57.5)	112 (60.9)	173 (59.7)	0.579
	High	45 (42.5)	72 (39.1)	117 (40.3)	
	Total	106 (100.0)	184 (100.0)	290 (100.0)	

^∗^Statistically significant.

**Table 4:** Logistic regression model.

Variables	*B*	SE	*p*	Odds ratio (CI)
Tumour differentiation	1.304	0.51	0.10	3.68 (1.36-9.93)
Inversion pattern				
POI II (ref)				1
POI III	1.086	0.59	0.06	2.96 (0.93-9.41)
POI IV	1.760	0.70	0.12	5.81 (1.46-23.09)
Constant	-3.158	0.88	<0.00	

## Data Availability

All data included here. Raw data can be provided upon request.
